# Sténose jéjunale néonatale par double diaphragme incomplete

**DOI:** 10.11604/pamj.2015.20.271.6471

**Published:** 2015-03-19

**Authors:** Imad El Biache, Youssef Bouabdallah

**Affiliations:** 1 Centre Hospitalier Universitaire Hassan II, Fès, Maroc

**Keywords:** Sténose, jéjunum, diaphragme, Stenosis, jejunum, diaphragm

## Image en medicine

Nous rapportons l'observation d'un nouveau né âgé de 27 jours qui présente depuis sa naissance un syndrome subocclusif intermittent fait de vomissements bilieux avec arrêt des matières sans arrêt des gaz. L'examen clinique trouvait un nourrisson dénutri et déshydraté avec un abdomen souple non distendu. La radiographie de l'abdomen sans préparation a montré des niveaux hydro aériques grêlo-coliques (A). L’échographie abdominale couplée au Doppler couleur n'a pas objectivé d'image en faveur de sténose hypertrophique du pylore ni en faveur du volvulus du grêle d'où la réalisation d'un TOGD qui a montré une importante dilatation gastrique, du cadre duodénal ainsi que de la première anse jéjunale faisant évoquer un diaphragme jéjunal incomplet (B). L'exploration chirurgicale a trouvé une disparité de calibre entre la première et la deuxième anse jéjunale avec découverte de deux diaphragmes incomplets intéressant la deuxième anse jéjunale (C). Nous avons procédé à la résection des deux diaphragmes avec suture de l'ouverture intestinale (D) et vérification de l’étanchéité. L’évolution clinique est bonne après 17 mois de recul.

**Figure 1:**
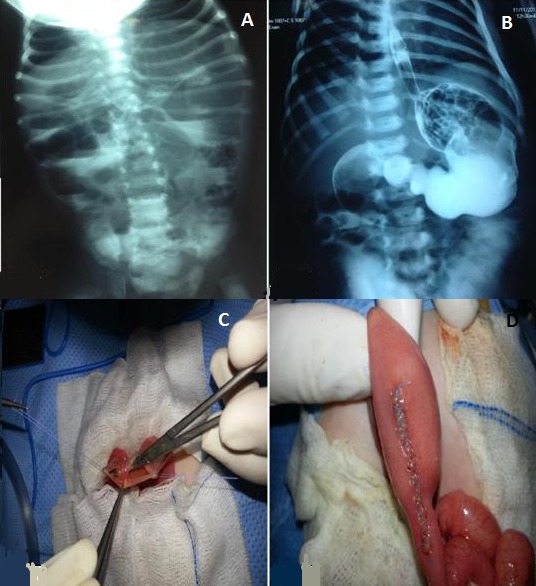
A) ASP debout montrant des niveaux hydro-aériques grêliques et coliques; B) TOGD objectivant une dilatation gastrique, du cadre duodénal ainsi que de la première anse jéjunale; C) découverte de deux diaphragmes incomplets intéressant la deuxième anse jéjunale; D) résection des deux diaphragmes avec suture de l'ouverture intestinale

